# The Overlooked Immune State in Candidemia: A Risk Factor for Mortality

**DOI:** 10.3390/jcm8101512

**Published:** 2019-09-20

**Authors:** Christian Ortega-Loubon, Beatriz Cano-Hernández, Rodrigo Poves-Alvarez, María Fe Muñoz-Moreno, Patricia Román-García, Sara Balbás-Alvarez, Olga de la Varga-Martínez, Esther Gómez-Sánchez, Estefanía Gómez-Pesquera, Mario Lorenzo-López, Eduardo Tamayo, María Heredia-Rodríguez

**Affiliations:** 1 Department of Cardiac Surgery, Clinic University Hospital of Valladolid, Ramon y Cajal Ave. 3, 47003 Valladolid, Spain; 2 BioCritic. Group for Biomedical Research in Critical Care Medicine, Ramon y Cajal Ave. 7, 47005 Valladolid, Spain; egp29@hotmail.com (E.G.-P.); mariolorenzo17@yahoo.es (M.L.-L.); eduardo.tamayo@uva.es (E.T.); maria_her_05@hotmail.com (M.H.-R.); 3 Department of Anaesthesiology, Clinic University Hospital of Valladolid, Ramon y Cajal Ave. 3, 47003 Valladolid, Spain; rodrigopoves@gmail.com (R.P.-A.); patyrgmp@hotmail.com (P.R.-G.); saritabalbas@hotmail.com (S.B.-A.); olga.v.m91@gmail.com (O.d.l.V.-M.); 4 Unit of Research, Clinic University Hospital of Valladolid, Ramon y Cajal Ave. 3, 47003 Valladolid, Spain; mfmunozm@saludcastillayleon.es; 5 Department of Surgery, Faculty of Medicine, University of Valladolid, Ramon y Cajal Ave 7, 47005 Valladolid, Spain

**Keywords:** lymphopenia, lymphocyte count, candidemia, mortality, prognosis, survival, immunosuppression

## Abstract

Lymphopenia has been related to increased mortality in septic patients. Nonetheless, the impact of lymphocyte count on candidemia mortality and prognosis has not been addressed. We conducted a retrospective study, including all admitted patients with candidemia from 2007 to 2016. We examined lymphocyte counts during the first 5 days following the diagnosis of candidemia. Multivariable logistic regression analysis was performed to determine the relationship between lymphocyte count and mortality. Classification and Regression Tree analysis was used to identify the best cut-off of lymphocyte count for mortality associated with candidemia. From 296 cases of candidemia, 115 died, (39.8% 30-day mortality). Low lymphocyte count was related to mortality and poor outcome (*p* < 0.001). Lymphocyte counts <0.703 × 10^9^ cells/L at diagnosis (area under the curve (AUC)-ROC, 0.783 ± 0.042; 95% confidence interval (CI), 0.700–0.867, *p* < 0.001), and lymphocyte count <1.272 × 10^9^ cells/L five days later (AUC-ROC, 0.791 ± 0.038; 95%CI, 0.716–0.866, *p* < 0.001) increased the odds of mortality five-fold (odds ratio (OR), 5.01; 95%CI, 2.39–10.93) at time of diagnosis, and three-fold (OR, 3.27; 95%CI, 1.24–8.62) by day 5, respectively. Low lymphocyte count is an independent predictor of mortality in patients with candidemia and might serve as a biomarker for predicting candidemia-associated mortality and poor outcome.

## 1. Introduction

Candidemia is a deadly infection found all over the world, accounting for 9% of all nosocomial bloodstream infection (BSI) [1,2]. This infection is associated with considerable morbidity and mortality, including prolonged hospital stays and increased healthcare costs, with 30% to 50% occurring in patients in the intensive care unit (ICU) [3]. Importantly, the crude candidemia mortality ranges from approximately 22% to 75%, despite advances in diagnosis and therapy [4,5,6,7,8]. Despite advancements such as effective broad-spectrum antifungals and recent management guidelines, the incidence of candidemia has doubled over the past two decades [7,9]. Studies in Italy and France have reported 1.73 to 6.7 cases per 1000 hospital admissions, while reports in Spain found a total incidence of 8.1 cases per 1000 admissions in a 2010 nationwide study [10,11]. Currently, candidemia is the fourth leading cause of BSI in hospitalized patients in the United States, the seventh leading cause in Europe, and the third in patients admitted to the ICU [7,12,13,14].

Various reports have thoroughly studied candidemia in both neutropenic and non-neutropenic patients [15,16,17,18]. Ma et al. identified neutropenia as an independent risk for mortality. The etiology in non-neutropenic patients is likely to be multifactorial. Contributing factors might be patients with severe comorbidities, invasive devices, complex procedures, total parenteral nutrition (TPN), broad-spectrum antibacterial agents, and aggressive immunosuppressive therapies [1,7,18,19].

Likewise, there are numerous reports regarding lymphopenia and poor outcome following sepsis [20,21,22]. Drewry et al. showed that persistent lymphopenia in patients with bacteremia and sepsis, even on the fourth day following a bacteremia diagnosis, predicts early and late mortality [21]. Similarly, Adrie demonstrated that persistent lymphopenia is a risk factor for acquired infections and mortality in patients admitted in the ICU [20]. Bacterial septic shock leads to immune system dysregulation, resulting in increased predisposition for candidemia [12,23]. Nonetheless, no reports have shown the direct repercussion of lymphocyte count at time of admission and its association with mortality and outcome in patients with candidemia.

Our study aimed to describe the characteristics between survivors and non-survivors in patients with candidemia and determine if lymphocyte count is an independent predictor of in-hospital mortality and impacts prognosis.

## 2. Materials and Methods

### 2.1. Study Subjects, Setting and Design

This study (ethics review number CINV 14–49) received full approval from the local Institutional Research Review Committee. The patient informed consent requirement was waived; however, all data were gathered anonymously from the hospital database for scientific purposes, in accordance with the Spanish law regulating personal privacy matters.

We conducted a retrospective observational study of computerized medical records of patients with positive blood cultures for Candida spp. at the Clinic University Hospital of Valladolid (Valladolid, Spain), an 800-bed tertiary care hospital, from January 2007 to December 2016. All patients admitted to the hospital with blood cultures positive for Candida were included. Patients were excluded if they were younger than age 18, had any prior immunodeficiency status that could alter our results (such as neutropaenia, human immunodeficiency virus or cancer), or had received immunosuppressive therapy.

### 2.2. Study Variables

Demographic data included age, sex, comorbidities, central venous catheter (CVC) placement, TPN, septic shock, prolonged mechanical ventilation (PMV) or renal replacement therapy (RRT). Candida colonization defined both by the Candida Score [24], and Ostrosky-Zeichner Score [25], and echinocandin treatment were collected as well. We recorded patient lymphocyte counts obtained during the first five days following candidemia diagnosis. Comorbidities and previously diagnosed underlying diseases were already established and reported in every patient’s medical record.

Candida score determines the likelihood of candidiasis, assigning one point each for TPN, abdominal surgery, and colonization and two points for sepsis [24]. The Ostrosky-Zeichner rule identifies broad-spectrum antibiotics (1 to 3 days), CVC placement (1 to 3 days), and those with at least 2 of the following risk factors: TPN (1 to 3 days), any type of dialysis (1 to 3 days), major surgery (−7 to 0 days), pancreatitis (−7 to 0 days), corticosteroids (−7 to 3 days) or other immunosuppressive agents (−7 to 0 days) [25].

The primary outcome was 30-day mortality. Secondary outcomes included the development of septic shock, PMV, and the requirement for RRT.

### 2.3. Definitions

An episode of candidemia was defined as the isolation of a Candida spp. from blood culture [3]. If there was more than 1 candidemia episode in the same patient, only the first episode was considered for the study.

Two blood culture sets from peripheral sites were obtained from patients with fever (≥38 °C) or clinical presentation suggestive of infection and sent off to the clinical laboratory of the Microbiology Department. The blood samples were analyzed using a BACTEC 9240 system (Becton-Dickinson Microbiology Systems, Franklin Lakes, NJ, USA) or BacT/Alert (BioMérieux SA, Marcy L’Etoile, France).

We defined septic shock using the international consensus definition for sepsis and septic shock as a subset of sepsis in which the underlying abnormalities of cellular and circulatory metabolism are profound enough to substantially increase mortality, in which vasopressor therapy is needed to elevate the mean arterial pressure ≥65 mmHg despite adequate fluid resuscitation [26,27]. PMV was considered greater than 48 h [28]. RRT was considered in case of potassium derangements, acid-base disbalance, fluid overload, or pronounced azotemia [29].

### 2.4. Statistical Analysis

Categoric variables are reported as percentages, and continuous variables are reported as the mean ± standard deviation or median (interquartile range) as appropriate, attending normal distribution. The assumption of normality was evaluated using the Shapiro–Wilk or Kolmogorov–Smirnov tests.

The associations between survivors and non-survivors of candidemia with other variables were identified using the chi-square or Fisher’s exact test (when the expected frequencies were <5) for categoric variables, and the Student’s *t*-test or the Mann-Whitney *U* test was used for continuous variables according to normality criteria.

Variables were included in univariate logistic regression analysis to derive 95% confidence intervals (CIs) for estimates. A multivariable logistic regression model was developed using a stepwise selection of predictors for 30-day mortality. Variables were included in the multivariable logistic regression model if the *p* value <0.1.

Regarding lymphocyte count, the optimal cut-off value with higher candidemia mortality was obtained using Classification and Regression Tree (CART) Analysis, which is ideally suited to the generation of clinical decision-making [30]. The ability of this cut-off value to predict 30-day candidemia mortality was further evaluated by using multivariate logistic regression analysis. Model calibration was assessed using the Hosmer-Lemeshow test.

Kaplan-Meier curves were plotted to show the 30-day survival probabilities according to lymphocyte counts.

All tests were 2-tailed. Odds ratio (OR) with 95% CI and *p*-values were reported. The level of significance was set at *p*  <  0.05. Data were analyzed using IBM SPSS Statistics for Windows version 24.0 software (IBM Corp, Armonk, NY, USA). CART-Analysis was done utilizing the *R* Software (Version 3.6.0. *R* Core Team, *R* foundation for Statistical computing, VIE, AU).

## 3. Results

### 3.1. Population Description

A total of 296 cases of candidemia were diagnosed from 2007 to 2016 (mean patient age, 63 ± 17) among non-neutropenic Mediterranean Caucasian patients (59.4% men) admitted to the hospital. Among them, 115 (38.9%) died within 30 days. Based on the 257,525 patients hospitalized over the 10-year period, the mean annual incidence of candidemia was 1.15 per 1000 admission. During their hospital stay, 146 (49.3%) patients had CVC placement, 123 (41.6%) needed PMV, 56 (18.9%) required RRT, and 82 (27.7%) received TPN.

Regarding underlying diseases, the proportions of patients with diabetes, cirrhosis, chronic obstructive pulmonary disease, heart failure (HF), renal failure, alcohol intake, dementia did not differ significantly between survivors and non-survivors. However, non-survivors were older, required more RRT, PMV, and developed more septic shock compared to survivors. Both Candida and Ostrosky scores were higher among non-survivors. Overall, only 122 (41.2%) cases received echinocandin treatment (Table 1).

**Figure 1:**
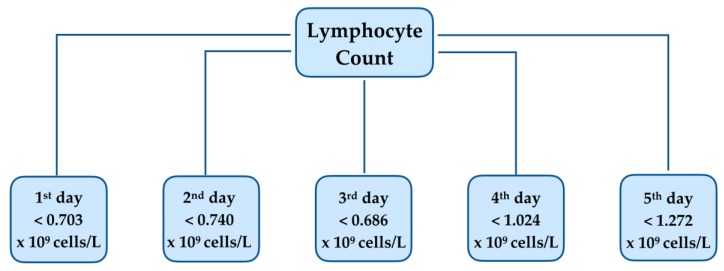
Decision tree generated by Classification and Regression Tree (CART) analysis, stratifying lymphocyte counts with a higher risk of mortality during the first days following the diagnosis of candidemia.

CART analysis identified a lymphocyte count <0.703 × 10^9^ cells/L at the time of diagnosis and <1.272 × 10^9^ cells/L five days afterwards as optimal cut-off value with higher risk of mortality after candidemia (Figure 1).

### 3.2. Impact of Lymphocyte Count on Mortality and Survival

At diagnosis of candidemia and by day 5, the absolute lymphocyte count was significantly higher in survivors (median, 1.058 × 10^9^ cells/L; range, 1.750 to 0.611 × 10^9^ cells/L) compared to the non-survivors (median, 0.858 × 10^9^ cells/L; range, 1.203 to 0.478 × 10^9^ cells/L; *p* 0.05; Table 1).

**Figure 2:**
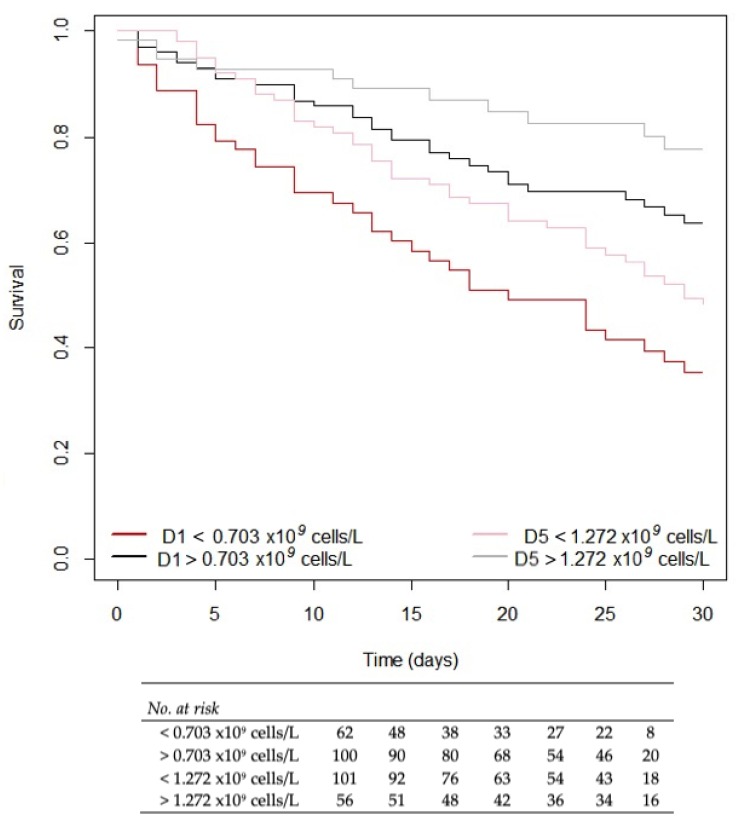
Survival patients with candidemia by lymphocyte count categorized by <0.703 × 10^9^ cells/L at diagnosis, <1.272 × 10^9^ cells/L 5 days later, and a comparison between each other.

Patients with lymphocyte counts >0.703 × 10^9^ cells/L at diagnosis and > 1.272 × 10^9^ cells/L at day 5 presented better survival rate. Patients with persistent low lymphocyte count who did not present a considerable rise of > 1.272 × 10^9^ cells/L by day five showed lower survival rate than those who had > 1.272 × 10^9^ cells/L by day five. Finally, patients with early onset of low lymphocyte count had the poorest survival rate (*p* < 0.001; Figure 2).

### 3.3. Univariate and Multivariable Logistic Risk Analysis for Mortality

Univariate regression analysis identified age (OR, 1.03; 95% CI, 1.02 to 1.05), PMV (OR, 2.73; 95% CI, 1.69 to 4.43), septic shock (OR, 2.95; 95% CI, 1.81 to 4.80), candida score (OR, 1.28; 95% CI, 1.13 to 1.45), as risk factors for mortality. Likewise, the cut-off values of lymphocyte count identified by CART analysis were significantly associated with mortality (Table 2).

Multivariable regression analysis confirmed PMV (OR, 3.07; 95% CI, 1.44 to 6.51), age (OR 1.49; 95% CI, 1.02 to 1.08), and low lymphocyte count as independent risk factors for mortality in patients with candidemia. At diagnosis, a lymphocyte count <0.703 × 10^9^ cells/L increases the odds of mortality 5.01-fold (OR, 5.01; 95% CI, 2.29 to 10.93). In addition, a lymphocyte count <1.272 × 10^9^ cells/L 5 days after diagnosis soars to a 3-fold risk (OR, 3.27; 95% CI, 1.24 to 8.62; *p* < 0.001) (Table 3).

**Figure 3:**
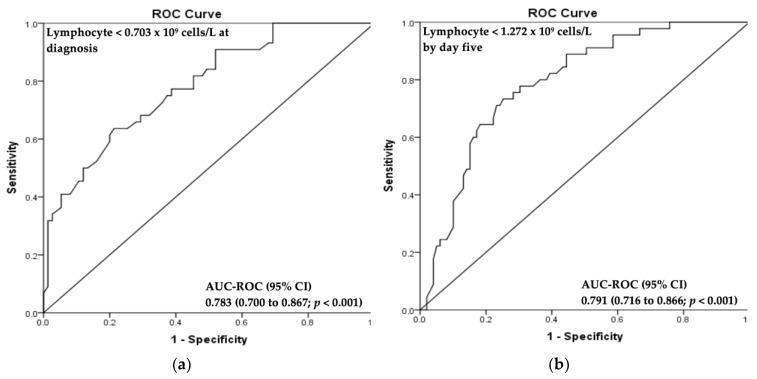
AUC-ROC Curve Analysis. Multivariable logistic regression model for lymphocyte count <0.703 × 10^9^ cells/L at diagnosis (**a**), and lymphocyte count <1.272 × 10^9^ cells/L by day 5 (**b**). Abbreviations: CI, confidence interval; AUC-ROC, area under the curve-receiver operating characteristic.

Combining these variables in a multivariable logistic model and including lymphocyte count, an AUC-ROC of 0.783 (95% CI, 0.700–0.867, *p* < 0.001) at diagnosis and AUC-ROC 0.791 (95% CI, 0.716–0.866, *p* < 0.001) 5 days later were obtained (Figure 3).

## 4. Discussion

This study found that persistent low lymphocyte count even 5 days after diagnosis of candidemia was an independent risk factor of 30-day mortality. Remarkably, multivariable regression analysis revealed that PMV, and age were also independent risk factors for mortality. The prognosis was better for cases with lymphocyte count > 0.703 × 10^9^ cells/L at diagnosis of candidemia and >1.272 × 10^9^ cells/L 5 days later.

An overall 30-day mortality rate of 38.9% was observed, which correlates with the broad mortality range of candidemia. Its mortality remains significantly high, especially in critically ill patients, reaching nearly 90% in patients with septic shock [31,32]. The differences in candidemia mortality rate can vary by ages, geographical area, medical management, and antifungal drug usage. Predictors for candidemia mortality were PMV, age and lymphocyte count. These results are consistent with previous studies such as Jia et al., Poves-Alvarez et al., and Sbrana et al., who identified these factors along with septic shock, chronic kidney disease, ascites, and concomitant bacterial infection as predictors of mortality [10,33,34]. Noticeably, outcome differed considerably according to age [35]. Ramos-Martinez et al. determined age as independently associated with mortality [36]. Advanced age, especially, is a leading risk factor for mortality in patients with candidemia [37]. Elderly patients presented higher mortality than younger patients and were more likely to receive inadequate antifungal treatment or even remain untreated [38]. Garnacho-Monterio et al., Kato et al., and Papadimitriou-Olivgeris et al. identified that appropriate empirical antifungal treatment was related to better prognosis [12,39,40]. Indeed, lack of inclusion of European Society of Clinical Microbiology and Infectious Disease (ESCMID) and Infectious Diseases Society of America (IDSA) guideline recommendations was an independent risk factor for a higher early and overall mortality [4]. In the same way, Keighley et al. found that age > 65 years, ICU admission, chronic organ dysfunction, preceding surgery within 30 days, haematological malignancy, source of candidaemia, and antibiotic therapy for ≥10 days were independent risk factors for candidemia mortality, which served to develop a risk predictive score [41]. Furthermore, Ma et al. and Kang et al. identified neutropenia, C. tropicalis, CVC, complicated abdominal surgery, and corticosteroids as poor prognosis factors [7,42].

Strikingly, multivariable logistic regression revealed lymphopenia as the most important risk factor for mortality at diagnosis of candidemia. The odds of dying of candidemia were up to 5.01 times higher when lymphocyte counts were <0.703 × 10^9^ cells/L at diagnosis. Drewry et al. demonstrated that day 4 absolute lymphocyte count was found to be independently associated with 28-day survival, and severe persistent lymphopenia (defined as an absolute lymphocyte count ≤0.6 × 10^9^ cells/L on the fourth day after sepsis diagnosis) was associated with increased development of secondary infections (*p* = 0.04) [21]. Furthermore, this study revealed that consistent low lymphocyte count, even on the fifth day after candidemia diagnosis, was related to increased mortality. The relationship between the immune system and candidemia has been studied by several authors. Toth et al. observed differences in the immune response between *C. albicans* and *C. parapsilosis*, which may serve to develop future immunotherapeutic strategies for these infections [43]. Gaffen et al. identified the paramount importance of cytokine IL-17 in the immune response against *Candida albicans* [44]. Unsinger et al. found that the treatment with IL-7 enhances the function of lymphocytes and improve candidemia outcome [45].

Following severe bacterial infections, such as sepsis, after an initial pro-inflammatory phase, an anti-inflammatory period occurs resulting in a prolonged period of the immunosuppressive stage called post-aggressive immunosuppression. This is due to a drop in the lymphocyte count as a consequence of circulating lymphocyte relocation to areas of infection, and sepsis-induced lymphocyte apoptosis [21]. This secondarily impaired immune response is correlated with poor outcomes [20]. Such severe persistent low lymphocyte count also predisposes a patient to secondary infections (e.g., a fungal infection), which in turn might explain its associated mortality, suggesting that patients who ultimately died did so due to candidemia-induced immunosuppression [21]. In fact, during a candida infection, an inflammatory response involving both pro-inflammatory and anti-inflammatory cytokines is produced and promotes a helper T cells 2 (TH2) response. The differentiation of lymphocytes to TH2 leads to the suppression of the immune response by different processes in which regulatory T lymphocytes participate. The downside of these regulatory cells has been studied in different scientific reports, in which both an immune activity suppression against *C*. *albicans* and a greater predisposition to candidemia have been demonstrated [46,47].

Prompt antifungal therapy is critical in the treatment of candidemia [39,48]. Cuervo et al. previously demonstrated that certain measures, such as early treatment and appropriate antifungal choice, have a strong impact on prognosis [4]. Indeed, the mortality for candidemia doubles if antifungal therapy is not initiated within the first 24 h of diagnosis [49,50,51]. The candida score and Ostrozky score can be helpful in excluding patients who are not likely to benefit from antifungal therapy rather than selecting those who will benefit from such therapy, thus restricting the irrational use of antifungal agents [51]. We followed the IDSA and ESCMID guidelines, which recommend the use of echinocandins for critically ill patients not previously exposed to azoles or infected with a non-albicans Candida spp as a result of a broader spectrum and greater efficacy than traditional antifungals [52,53]. Poves-Alavrez et al., conversely, found that 30-day mortality was not modified by antifungal treatment [10]. Also, this study demonstrates that antifungal prophylaxis or specific treatment may not be enough. Despite all these measures, the overall incidence and morbidity and mortality associated with candidemia remain strikingly high. Therefore, this BSI should be approached from a different perspective. Addressing the early derangement of the immune system may be necessary, and immune-adjuvant therapy should be offered. Potential immunotherapeutic agents such as interleukin-7 or anti-programmed cell death-1 antibody may act to increase CD4 and CD8 T cell production, block lymphocyte apoptosis, and prevent T cell fatigue. The management of candidemia would be more effective if these therapies were administered to patients with evidence of low lymphocyte count and high risk for in-hospital mortality [21].

In terms of feasibility, considering the standarised laboratory lymphocyte cut-off value and that persistently low levels of circulating lymphocytes following the diagnosis of candidemia independently predicts survival, lymphocytes may serve as a useful clinical biomarker for candidemia-induced immunosuppression. Prolonged low lymphocyte count is a candidate marker of persistent immunosuppression in septic patients, and absolute lymphocyte counts are easily measured during routine care. Thus, lymphocyte counts are very suitable for clinical application in busy departments at times of candidemia suspicion, and the routinely measured total lymphocyte count may be considered. Lymphocyte count might be used as a simple and reproducible marker of post-aggressive immunosuppression [20]. Furthermore, use of lymphocytes as biomarkers may help prevent the overuse of antifungal agents, given the poor prognosis related to lymphocyte count <0.703 × 10^9^ cells/L. Finally, they may serve to evaluate patient response to antifungal therapy.

### Study Limitations

Given this is an observational-retrospective study, there is a potential for confounding factors because accuracy relies on adequate hospital coding. Thus, this study was prone to a possible underestimation of the real number of cases and misclassification. In addition, the timing of the antifungal agent should be recorded. Despite these limitations, and although risk factors associated with candidemia mortality are well known, this study addresses the relationship between lymphocytopenia and candidemia mortality, which was largely unexplored to date.

## 5. Conclusions

Persistent low lymphocyte count is an independent predictor of mortality in patients with candidemia. A lymphocyte count <0.703 × 10^9^ cells/L at diagnosis or <1.272 × 10^9^ cells/L even 5 days later indicate a poor prognosis with nearly five-fold and three-fold increases in 30-day mortality.

## Figures and Tables

**Table 1:** Clinical characteristics of patients with candidemia by mortality.

Variable	Total (*n* = 296)	Survivors (*n* = 181)	Non-Survivors (*n* = 115)	*p*-Value
Age	63 ± 17.4	60.0 ± 19.0	68.0 ± 13.0	<0.001
Sex (% male)	177 (59.4)	102 (56.4)	74 (64.3)	0.172
**Comorbidities**				
Alcohol intake	23 (7.8)	12 (6.6)	11 (9.6)	0.358
COPD	33 (11.2)	20 (11.0)	13 (11.3)	0.972
Diabetes	61 (20.6)	32 (17.7)	29 (25.2)	0.124
Renal Disease	49 (16.6)	28 (15.5)	20 (17.4)	0.662
Cirrhosis	10 (3.4)	5 (2.8)	5 (4.3)	0.518
HF	56 (18.9)	29 (16.0)	27 (23.5)	0.110
Dementia	6 (2.0)	5 (2.8)	1 (0.9)	0.260
**Hospital Admission**				
**Source Infection**				
CVC	146 (49.3)	81 (44.8)	65 (56.5)	0.004
Abdominal	30 (10.1)	15 (8.3)	15 (13.0)	
TPN	82 (27.7)	35 (19.3)	46 (40.0)	<0.001
Other	120 (40.5)	85 (47.0)	35 (30.4)	
Surgery	126 (42.6)	72 (39.8)	54 (47.0)	0.223
PMV	123 (41.6)	58 (32.0)	65 (56.5)	<0.001
RRT	56 (18.9)	28 (15.5)	28 (24.3)	0.060
Septic Shock	146 (49.3)	70 (38.7)	75 (65.2)	<0.001
Candida Score	2.0 ± 1.9	2.0 ± 1.8	3.0 ± 1.9	<0.001
Ostrosky Score	2.4 ± 2.2	1.9 ± 2.0	3.0 ± 2.2	<0.001
Lymphocyte count at diagnosis, ×10^9^ cells/L, median (IQR)	0.952 (1.588–0.462)	0.998 (1.746–0.535)	0.778 (1.364–0.410)	0.045
Lymphocyte count by day 2, ×10^9^ cells/L, median (IQR)	0.900 (1.688–0.480)	0.979 (1.745–0.543)	0.680 (1.372–0.404)	0.038
Lymphocyte count by day 3, ×10^9^ cells/L, median (IQR)	0.935 (1.567–0.452)	1.058 (1.614–0.613)	0.747 (1.450–0.310)	0.010
Lymphocyte count by day 4, ×10^9^ cells/L, median (IQR)	0.922 (1.633–0.513)	1.078 (1.803–0.581)	0.753 (1.211–0.418)	0.011
Lymphocyte count by day five, ×10^9^ cells/L, median (IQR)	0.947 (1.588–0.526)	1.058 (1.750–0.611)	0.858 (1.203–0.478)	0.050
Echinocandins	122 (41.2)	76 (42.0)	46 (40.0)	0.735
**Causative Organism**				
*C. albicans*	179 (60.4)	96 (62.3)	83 (58.5)	0.905
*C. parapsilosis*	40 (13.5)	21 (13.6)	17 (12.0)	0.525
*C. glabrata*	36 (12.5)	20 (13.0)	16 (11.3)	0.621
*C. tropicalis*	29 (9.8)	11 (7.1)	18 (12.7)	0.076
*C. lusitaniae*	12 (4.1)	4 (2.6)	8 (5.6)	0.254

Values are expressed absolute number (percentage) and mean (standard deviation). Statistical significance was defined as *p* < 0.05. Abbreviations: COPD, chronic obstructive pulmonary disease; CVC, central venous catheter; HF, heart failure; IQR, interquartile range; PMV, prolonged mechanical ventilation; RRT, renal replacement therapy; TPN, total parenteral nutrition.

**Table 2:** Univariate regression analysis for mortality associated with candidemia.

	Univariate Analysis	
	OR (95% CI)	*p*-Value
Age	1.030 (1.01–1.05)	<0.001
PMV	2.734 (1.69–4.43)	<0.001
Septic shock	2.946 (1.81–4.80)	<0.001
Candida Score	1.282 (1.13–1.45)	<0.001
Lymphocyte count (diagnosis) (<0.703 × 10^9^ cells/L)	3.11 (1.62–5.98)	0.001
Lymphocyte count (day 2) (<0.740 × 10^9^ cells/L)	2.108 (1.16–3.83)	0.014
Lymphocyte count (day 3) (<0.686 × 10^9^ cells/L)	2.213 (1.23–3.97)	0.008
Lymphocyte count (day 4) (<1.024 × 10^9^ cells/L)	2.737 (1.40–5.36)	0.003
Lymphocyte count (day 5) (<1.272 × 10^9^ cells/L)	3.435 (1.60–7.38)	0.002

Abbreviations: CI, confidence interval; HF, heart failure; OR, odds ratio; PMV, prolonged mechanical ventilation.

**Table 3:** Multivariable regression analysis for mortality associated with candidemia using lymphocytes as dichotomous variable.

	Multivariable Analysis at Diagnosis		Multivariable Analysis at Day 5	
	OR (95% CI)	*p*-Value	OR (95% CI)	*p*-Value
PMV	3.07 (1.44–6.51)	0.004	3.98 (1.77–8.95)	0.001
Age	1.49 (1.02–1.08)	0.001	1.05 (1.01–1.08)	0.012
Lymphocyte count (diagnosis) < 0.703 × 10^9^ cells/L	5.01 (2.29–10.93)	0.002		
Lymphocyte count (day 5) < 1.272 × 10^9^ cells/L			3.27 (1.24–8.62)	0.016

Hosmer-Lemeshow at diagnosis χ^2^_8_ = 5.011; *p* = 0.756. Hosmer-Lemeshow at day 5 χ^2^_8_ = 3.786; *p* = 0.876. Abbreviations: CI, confidence interval; OR, odds ratio; PMV, prolonged mechanical ventilation.
